# P035: Costs of illness analysis of community and hospital acquired gastroenteritis in a small South Indian town

**DOI:** 10.1186/2047-2994-2-S1-P35

**Published:** 2013-06-20

**Authors:** S Parimalakrishnan, A Anton Smith, GP Mohanta, PK Manna

**Affiliations:** 1 Pharmacy, Annamalai University, Annamalai Nagar, India

## 

Occurrences of gastroenteritis have been increasing and become a threat to public health. While the morbidity of rotavirus gastroenteritis are extensive in developing countries like India; the date on the cost of illness are not easily available. The present study was aimed to analyze and compare the expenditure incurred during community and hospital acquired rotavirus and norovirus gastroenteritis in the paediatric and elder patient populations. The present study was a prospective epidemiologic study conducted in selected rural areas of Chidambaram, Tamil Nadu, India. Cost of illness was calculated as average cost per episode per case in ambulatory setup.

Rotavirus and norovirus were identified in 42 of the 68 and 34 out of 79 in stool samples tested of paediatric and elder patients respectively. Patients mean age was 18.36 ± 1.35 months and 65.88 ± 4.71 years in paediatric and elder patients respectively. 7 patients were admitted to hospital due to severe condition. The average period of fever was observed as 2.1 and 1.9 days while vomiting was present for 1.6 and 1.4 days and diraahoea was for 3.1 and 2.7 days in paediatric and elder patients respectively. The total cost per episode varied from ₹87 ± 1.55 to ₹309 ± 20.01 and from ₹112 ± 3.33 to ₹459 ± 23.27 in the ambulatory setting for paediatric and elder patients respectively. The total cost includes direct, direct nonmedical and indirect costs. The majority of hospital-related costs were paid out of their pocket. No one was reimbursed by health insurance payers. The average workdays lost by parents, guardians and other relatives varied between among study population care givers and settings, ranging from 1.7 to 5.3 days and this was the significant cost spent. From the result of the present study we conclude that in infants regular vaccination for rotavirus may improve immunity and decrease cost of illness whereas in elder patients affected by gastroenteritis, sufficient improvement in quality of life could be achieved by increasing funds in health sector.

## Disclosure of interest

None declared

